# Daily Steps After Hip Fracture in Older Adults and Their Relationship with Functional Recovery

**DOI:** 10.3390/jcm14113906

**Published:** 2025-06-02

**Authors:** Cristina Bermejo Boixareu, Leticia Pecharroman Anton, Laura Mateos del Amo, Manuel Benito Fuentes, Gema Piña Delgado, Macarena Díaz de Bustamante de Ussía, Ana Royuela Vicente, F. Javier Martín-Sánchez, Rosa M. Martínez Ruiz, Carlos Fagundez García, Rafael Bielza Galindo, Verónica García Cárdenas, Clara Valverde Fontcuberta, Cristina González de Villaumbrosía, Marta I. Sanz-Pérez, Jesús Campo Loarte

**Affiliations:** 1 Geriatrics Department, University Hospital Puerta Hierro Majadahonda, 28222 Madrid, Spain; leticia.pecharroman@salud.madrid.org (L.P.A.); mbfuentes@salud.madrid.org (M.B.F.); gema.pina@salud.madrid.org (G.P.D.); macarena.diaz@salud.madrid.org (M.D.d.B.d.U.); 2 Biostatistics Unit, Hospital Universitario Puerta de Hierro Majadahonda, IDIPHISA. CIBERESP, ISCIII., 28222 Madrid, Spain; aroyuela@idiphim.org; 3 Functional Recovery Unit, Hospital de Emergencias Enfermera Isabel Zendal, 28055 Madrid, Spain; fmartins@salud.madrid.org; 4 Geriatrics Department, University Hospital Infanta Sofía, 28702 Madrid, Spain; rmartinezr@salud.madrid.org (R.M.M.R.); carlos.fagundez@salud.madrid.org (C.F.G.); rafael.bielza@salud.madrid.org (R.B.G.); 5 Geriatrics Department, University Hospital Rey Juan Carlos, 28993 Madrid, Spain; veronica.garciac@hospitalreyjuancarlos.es (V.G.C.); clara.valverde@hospitalreyjuancarlos.es (C.V.F.); cristina.gonzalez@hospitalreyjuancarlos.es (C.G.d.V.); 6 Orthopaedic Surgery and Traumatology Department, University Hospital Puerta Hierro Majadahonda, 28222 Madrid, Spain; martaisabel.sanz@salud.madrid.org (M.I.S.-P.); jesus.campo@salud.madrid.org (J.C.L.)

**Keywords:** hip fracture, daily steps, physical activity, functional recovery, older adults

## Abstract

**Background**: Step count has emerged as an objective indicator of physical activity, yet its association with functional recovery following hip fracture remains unclear. **Objective**: This study aimed to evaluate daily step counts after hospital discharge in older adults with hip fracture and to determine thresholds associated with functional improvement. **Methods**: A prospective, observational study was conducted in patients aged over 75 years admitted with hip fracture. Daily steps were recorded using validated activity trackers. Functional status was assessed at one, three, and six months after discharge through telephone interviews. Functional improvement was defined as an increase of at least 5 points on the Barthel Index. Step count thresholds were estimated using Liu’s method based on receiver operating characteristic (ROC) analysis. **Results**: Ninety-four patients were included with a mean age of 83.2 ± 6 years, with 80.8% being female. Of the patients included in the study who recorded their daily steps after hip surgery, 59 patients (72%) improved during the first month after discharge with a median of 220 daily steps (IQR: 103.5–494.5; cut-off point at 150 steps). At the third month after hip fracture, 77 patients (86.5%) showed functional improvement with a median of 778 steps (IQR: 263–1697; cut-off point at 425 steps). At month six, 65 patients (80.2%) showed functional improvement with a median of 1757 steps (IQR: 696–3388; cut-off point at 2404 steps). **Conclusions**: In older adults discharged after hip fracture, achieving more than 150 steps per day in the first month, 425 steps in the third month, and 2404 steps by the sixth month was associated with functional improvement on the Barthel Index. These findings suggest potential activity targets to support mobility recovery. However, further research is warranted to confirm these associations in larger and more diverse populations. These thresholds, although exploratory, may also assist clinicians in monitoring and identifying patients at risk of poor functional recovery.

## 1. Introduction

Functional decline is a major consequence of aging and is projected to double in the coming years [1,2], with substantial implications for autonomy, quality of life, and survival [3]. In particular, hip fractures represent one of the most disabling acute events in older adults, with over 50% of patients experiencing new dependence and nearly 30% mortality within the first year [4].

Functional recovery after hip fracture is critical for restoring independence in activities of daily living and avoiding institutionalization. Early mobilization and physical activity have been associated with better outcomes. However, there is limited evidence defining specific physical activity thresholds that predict successful recovery.

In the review by Tudor-Locke et al. [5,6], activity levels in healthy older adults were defined based on the number of steps taken per day: sedentary (<5000 steps/day), low active (5000–7499 steps/day), somewhat active (7500–9999 steps/day), active (≥10,000 steps/day), and highly active (>12,500 steps/day). However, these thresholds cannot be extrapolated to the entire older population.

Previous studies have shown that step counts are associated with important health outcomes in older adults. Walking fewer than 4000 steps per day has been linked to increased risks of fractures, depression, and frailty [7,8], while walking more than 6000–8000 steps per day has been associated with lower mortality [9,10,11,12]. In hospitalized older adults with acute illnesses, reduced step counts during admission have been related to hospital-acquired disability, readmission, and poor discharge outcomes, with thresholds between 400 and 900 steps per day showing predictive value [13,14].

In patients hospitalized specifically for hip fracture, recorded step counts during the inpatient period are extremely low, typically around 35–80 steps per day [15,16]. Despite the high disability burden after hip fracture, no prior studies have established post-discharge daily step thresholds associated with meaningful functional recovery, such as improvements in the Barthel Index.

Although step count has been studied in hospitalized older adults with acute illnesses and during hospitalization for hip fracture, a critical gap remains regarding post-discharge activity levels. Existing studies have primarily reported very low step counts during inpatient stays, but none have established specific post-discharge step targets linked to functional recovery outcomes.

Fitbit devices or pedometers in older adults are a successful, easy, and inexpensive motivational tool to increase ambulatory activity and have shown benefits in functional status [17,18,19]. Functional recovery, defined as the regaining of independence in basic activities of daily living, is crucial after hip fracture, as it determines long-term autonomy, reduces the risk of institutionalization, and improves survival. Given the high rates of disability and mortality following hip fractures, defining ambulatory goals that directly predict functional improvement is essential.

This study addresses this gap by identifying clinically daily step thresholds that are associated with significant improvements in functional status (reflected by gains in the Barthel Index) during the first 6 months following discharge.

## 2. Materials and Methods

A prospective observational study was conducted in three different tertiary hospitals with 296, 358, and 613 beds, respectively.

Inclusion criteria were patients over 75 years of age who were admitted for a hip fracture to one of the hospitals participating in the study, and who were able to ambulate independently, with or without technical assistance, prior to admission.

Patients were excluded if they required immobility after surgery, declined to provide informed consent, or were dependent on personal assistance for ambulation prior to admission. Additionally, patients with a baseline Barthel Index score below 30 were also ruled out, as such a level of functional dependence typically indicates non-ambulatory status, rendering step count monitoring irrelevant for assessing mobility progress. Patients with advanced dementia, defined as more than 5 errors on the Pfeiffer Short Portable Mental Status Questionnaire (SPMSQ), were also excluded. These patients are generally unable to reliably engage with physical activity monitoring, often lack the capacity to provided informed consent, and critically are unlikely to understand or adhere to step count-based mobility goals.

Clinical assessments were conducted at admission, at discharge, and at the first, third, and sixth months after discharge. Functional status was evaluated using the Barthel Index, cognitive status was assessed using the SPMSQ, nutritional status was screened using the Mini Nutritional Assessment (MNA) short form, frailty was measured with the FRAIL questionnaire, and muscle strength was evaluated using handgrip strength. Additional variables recorded included preoperative time, length of hospital stay, readmission rates, and mortality.

Daily step counts were recorded using Xiaomi Mi Band devices, which have demonstrated acceptable validity for step counting in older adults under free-living conditions [20,21,22]. Studies have shown good agreement between these devices and research-grade accelerometers; therefore, no additional validation against gold-standard methods was performed in this study. The Xiaomi device was selected for its low cost, ease of use, and accessibility, reflecting real-world conditions under which step count-based mobility goals would realistically be implemented.

The number of steps was recorded daily, and weekly median step counts were then calculated from these daily recordings. Potential inaccuracies in step detection, particularly at very low walking speeds or among individuals using assistive devices, should be considered when interpreting the results.

The study was registered and approved by the hospital ethics committees of each hospital (code PI 55/19). Approval was granted before enrollment of the first patient: University Hospital Puerta de Hierro Majadahonda ethics committee (25 September 2019), University Hospital Rey Juan Carlos ethics committee (5 September 2022), and University Hospital Infanta Sofía ethics committee (24 March 2022). The study was conducted in accordance with the Declaration of Helsinki.

### Statistical Analysis

A descriptive analysis was performed for categorical variables using absolute and relative frequencies, and for numerical variables using the mean and standard deviation or the median and interquartile range (25th and 75th percentiles), according to compliance with the assumption of normality. To estimate the cut-off points for the number of daily steps associated with functional improvement at the first, third, and sixth months after hip fracture, a clinically relevant improvement in functional status was defined as an increase of at least five points on the Barthel Index [23]. The relationship between step counts and clinical outcomes such as falls, hospital readmission, and mortality was explored using correlation analysis, logistic regression, and ROC curve analysis. Cut-off thresholds for step counts were selected using Liu’s method [24], which identifies the point that maximizes the product of sensitivity and specificity based on ROC analysis. Due to distinct surgical treatments and recovery patterns, we performed a subgroup analysis comparing intracapsular fractures (treated with arthroplasty) and extracapsular fractures (treated with osteosynthesis). The level of significance was set at 0.05. The statistical package used was Stata/IC v.15.1. (StataCorp. 2017. Stata Statistical Software: Release 15. College Station, TX: StataCorp LLC, Texas, USA).

## 3. Results

**Figure 1:**
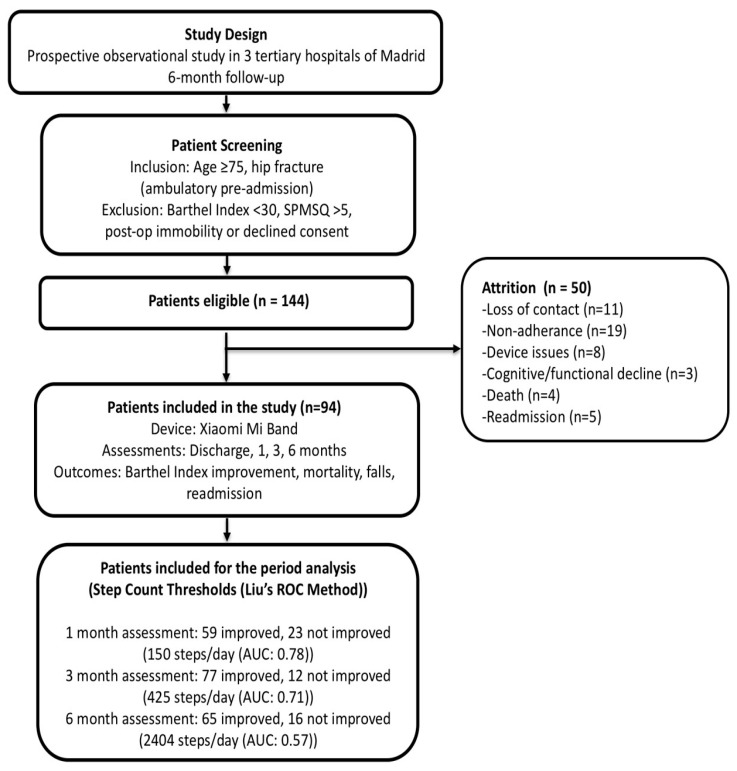
A flow chart illustrating the study process.

Prior to discharge, 144 patients were enrolled in the study, and daily step count data were successfully collected from 94 patients (67 patients from the 613-bed hospital, 10 from the 352-bed hospital, and 17 from the 296-bed hospital). Attrition occurred due to loss of contact (n = 11), non-compliance with wearing the activity bracelet (n = 19), lack of family support for device use (n = 8), functional and/or cognitive decline (n = 3), death (n = 4), or hospital readmission (n = 5) (Figure 1). Withdrawn patients did not differ significantly from those included in terms of baseline characteristics, so no selection bias is expected. The reasons for discontinuation of step count monitoring were unrelated to improvements in Barthel Index score (Table 1).

The mean age of the sample was 83.2 ± 6 years, and 80.8% of participants were female. Baseline characteristics are shown in Table 1. The Charlson Comorbidity Index had a median score of 2 points (IQR: 1–4). Median handgrip was 16 kg (IQR: 12–18) and the median BMI was 24 kg/m^2^ (IQR: 21.8–27.4). The median score on the short version of the MNA was 11 points (IQR: 9–12).

The median preoperative hospital stay was 2 days (IQR: 1–3), and 84% of patients were lifted to a sitting position within the first 24 h after surgery. The median length of stay was 6.5 days (IQR: 5–10). At discharge, 83% of patients returned home, 7.4% were transferred to nursing homes, and 9.6% were transferred to functional recovery units. The median Barthel Index score at discharge was 65 (IQR: 60–70) and 60.6% of patients were receiving nutritional supplements.

**Figure 2:**
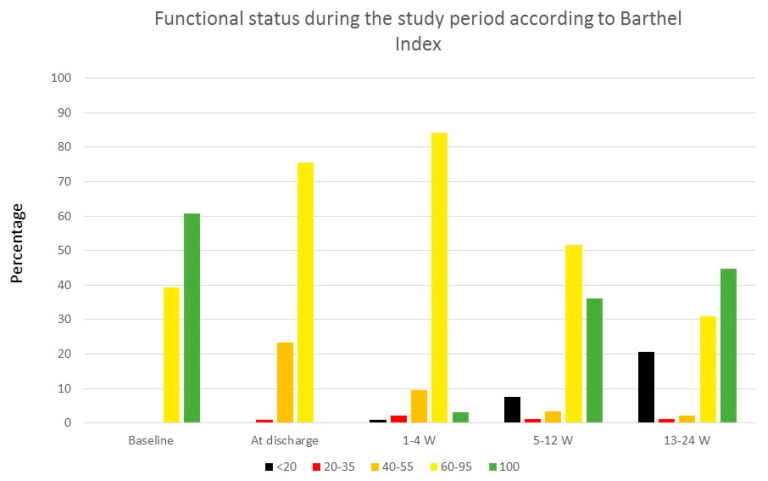
Functional status of the sample during the study period. Functional status of patients according to the Barthel Index across the different phases of the study.

**Figure 3:**
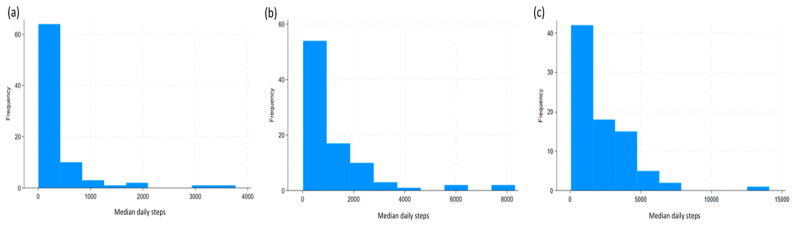
(**a**) Distribution of steps counts at 1 month; (**b**) Distribution of steps counts at 3 months; (**c**) Distribution of steps counts at 6 months.

Figure 2 displays changes in functional status based on Barthel Index during the study period. Figure 3 illustrates the distribution of median daily steps during the first, third, and sixth months of follow-up.

**Figure 4:**
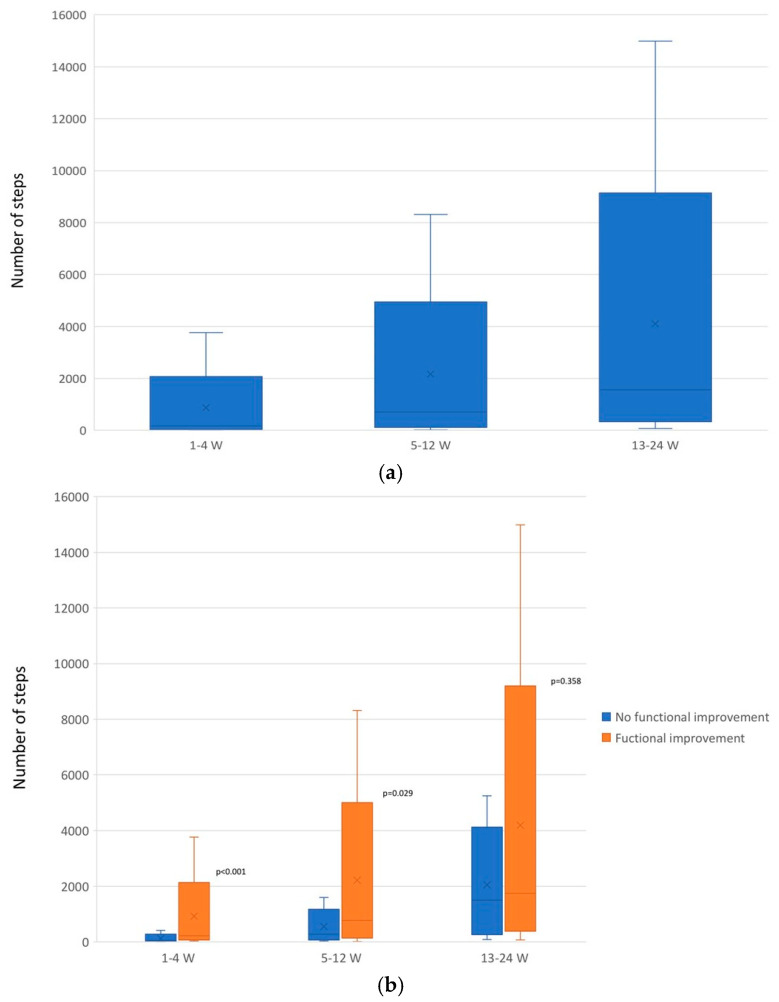
(**a**) Range of daily step counts in older adults with hip fracture across the different phases of the study (median and interquartile range). (**b**) Range of daily step counts in older adults with hip fracture, stratified by functional improvement status, across different phases of the study (median and interquartile range).

**Figure 5:**
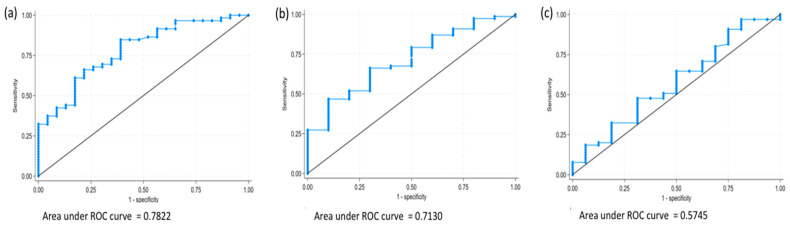
(**a**) ROC curve for predicting daily step counts at 1 month; (**b**) ROC curve for predicting daily step counts at 3 months; (**c**) ROC curve for predicting daily step counts at 6 months.

One month after hip fracture, 72% of patients showed a functional improvement of at least five points on the Barthel Index. The median Barthel Index score was 82.5 (IQR: 65–90), with a median improvement of 17.5 points (IQR: 5–25). Among the 59 patients who improved, the median daily step count was 220 steps (IQR: 103.5–494.5), and the group that did not improve had a median of 61.5 steps (IQR: 46–149.5) (*p* < 0.001) (Figure 4a,b). After adjusting for age, comorbidity, and previous functional status, the association between the number of steps and improvement in Barthel score was maintained (*p* = 0.039). The area under the ROC curve was 0.78 (Figure 5). According to Liu’s method, the optimal cut-off point was identified at 150 daily steps, with a sensitivity of 66.1% (95% CI 52.6–77.9%) and a specificity of 78.3% (95% CI 56.3–92.5%). Clinically, patients achieving a median of 220 steps per day showed greater functional improvement compared to those with lower step counts. A threshold of 150 steps per day was associated with a clinically meaningful gain of at least 5 points on the Barthel Index. This level of activity likely reflects basic indoor ambulation, such as moving independently between rooms at home.

During the first month, five patients died, three patients were readmitted due to upper gastrointestinal bleeding, hyponatremia, and a new fracture. One patient reported having had a fall. At one month, 7.5% of patients were still in functional recovery units, 8.6% were in nursing homes, and the reminder lived at home.

At the third month after hip fracture discharge, the median Barthel Index was 90 points (IQR: 85–100), with a median improvement of 30 points compared to discharge (IQR: 25–35). Among the 89 patients with recorded steps, 77 showed functional improvement with a median of 778 daily steps (IQR: 263–1697); those who did not improve had a median of 277 steps per day (IQR: 110–747) (*p* = 0.029) (Figure 4a,b). The area under the curve was 0.713 (Figure 5). The cut-off point was established at 425 daily steps with a sensitivity of 66.2% (95% CI 54.6–76.6%) and a specificity of 70% (95% CI 34.8–93.3%). At three months, a threshold of 425 steps per day was associated with further functional gains. During this period, two patients experienced falls (one fell twice), three patients were hospitalized, and one of them died.

At sixth months, median Barthel Index was 95 (IQR: 65–100) with a median improvement of 30 points (IQR: 10–35), and 0 points from baseline (IQR: −20–0). During this period, 81 patients had recorded steps, of which 65 patients showed functional improvement with a median of 1757 steps per day (IQR 696–3388); the 16 patients who did not present functional improvement had a median of 1502 daily steps (IQR: 452.5–3000) (*p* = 0.35) (Figure 4a,b). The area under the curve was 0.57 (Figure 5) and the cut-off point was established at 2404 steps per day with a sensitivity of 47.7% (95% CI 35.1–60.5%) and a specificity of 68.8 (95% CI 41.3–89.0%). This step level may reflect the ability to perform more advanced activities of daily living, including community mobility, indicating more complete recovery of pre-fracture function. The median improvement in Barthel Index after discharge was 30 points (IQR: 10–35) and the median improvement since admission was 0 points (IQR: −20–0). At this time, two patients documented 1–2 falls and there were four hospital readmissions and two deaths.

A subgroup analysis was conducted to examine differences in recovery between patients with intracapsular fractures treated with arthroplasty and those with intertrochanteric or subtrochanteric (extracapsular) fractures treated with osteosynthesis. In the first month after discharge, the empirical cut-off point for daily step count in the intracapsular fracture group was estimated at 148 steps (AUC: 0.81; sensitivity: 77%; and specificity: 86%). For the extracapsular fracture group, the cut-off was 75 steps per day (AUC: 0.76; sensitivity: 83%; and specificity: 69%). Between the second and third month, patients treated with arthroplasty (intracapsular fractures) had a cut-off point of 217 steps per day (AUC: 0.70; sensitivity: 91%; and specificity: 50%), while those treated with osteosynthesis (extracapsular fractures) had a higher threshold of 433 steps per day (AUC: 0.80; sensitivity: 60%; and specificity: 100%). From the third to sixth month, the cut-off point for older adults with intracapsular fractures was 1263 daily steps (AUC: 0.65; sensitivity: 73%; and specificity: 57%). For those with extracapsular fractures, it was 2364 steps per day (AUC: 0.56; sensitivity: 46%; specificity: 67%).

## 4. Discussion

Our findings show that walking a minimum number of daily steps early after hip fracture discharge is statistically associated with functional improvement and may also have meaningful clinical relevance. Achieving more than 150 steps per day in the first month, 425 per day steps by the third month, and 2404 per day steps by the sixth month likely represents a threshold of mobility and independence that may help prevent long-term functional decline.

These cut-offs may offer preliminary activity targets: for example, walking more than 150 steps daily soon after discharge likely reflects the ability to perform essential indoor movements (e.g., getting to the bathroom, kitchen, and bed), which is critical for maintaining autonomy and reducing caregiver burden. Exceeding 425 steps per day by three months suggests greater outdoor mobility and basic community participation, while reaching 2404 steps per day by six months may correspond to regaining more advanced activities of daily living, thereby reducing the risk of institutionalization and supporting social reintegration.

The number of steps recorded in our study is comparable to those published in other studies of hip fracture patients during hospitalization [15,16]. Older adults with hip fracture take significantly fewer steps [13,14] than patients hospitalized for other acute medical conditions [25,26,27,28].

Previous research has shown that walking more than 275 steps per day during hospitalization was a better predictor of the risk of hospital readmission within the first month than basic activities of daily living [26]. However, in our study, post-discharge step counts were not significantly associated with mortality or readmission rates. Nonetheless, we found a significant association between the number of steps after discharge and functional decline at one month. This supports prior observations that lower mobility levels after hospitalization are associated with worse functional outcomes [29].

Step count represents a simple, objective measure of physical activity that could serve as a motivational target for older adults to achieve functional recovery. Investigation into physical activity in the geriatric population is essential both during hospitalization and after hospital discharge [26], especially following disabling events such as hip fracture. However, further studies are needed to determine specific goals and thresholds for optimal outcomes. For example, in the sub-analysis by type of hip fracture, or type of intervention; in the first month, patients with intracapsular fractures showed higher functional recovery with relatively fewer steps, possibly due to earlier mobilization permitted by joint replacement and fewer post-operative restrictions [30]. From the third month onward, extracapsular fracture patients appeared to require more steps for equivalent functional recovery, possibly reflecting delayed early progress but steeper recovery potential once bone healing stabilizes. These subgroup findings should be interpreted cautiously due to the limited sample size.

Furthermore, defining step count goals may have practical clinical applications by offering patients and caregivers a simple, objective measure to monitor recovery progress at home. Such mobility targets could complement traditional interventions by encouraging unsupervised daily activity and maintaining engagement between therapy sessions. Developing patient-centered strategies that promote safe, incremental mobility could be critical for improving outcomes after hip fracture. These thresholds may also assist clinicians in monitoring and identifying patients at risk of poor functional recovery.

Although conventional activity monitors, such as Fitbit or Xiaomi Mi Band devices, offer an accessible means to track mobility, their accuracy may be limited, particularly during early recovery phases when patients use walking aids and exhibit altered gait patterns [19]. Nevertheless, such devices remain valuable for setting practical goals and promoting functional stimulation in older adults, as they have demonstrated reasonable validity [19,20,21,22,31].

## 5. Limitations

Several methodological limitations should be considered when interpreting our findings. First, although the Xiaomi Mi Band has demonstrated acceptable validity for step counting in older adults [20,21,22], as a consumer-grade device, it may have limited accuracy, particularly at low walking speeds or when assistive devices (i.e., walkers or crutches) are used [19]. This could affect the precision of step counts, especially during the early recovery phase. More accurate devices exist but are costly and less accessible, limiting their feasibility for widespread clinical use. Thus, we used commercially available models to ensure the applicability of our results to real-world practice.

Second, the study had a relatively high dropout rate, with 35% of initially enrolled participants lost to final analysis. Although baseline characteristics were similar between completers and non-completers, the possibility of selection bias cannot be excluded.

Third, the exclusion of patients with advanced dementia or severe baseline functional dependence (Barthel Index < 30) limits the generalizability of our findings. The clinical and demographic characteristics of our sample are similar to those reported in the Spanish National Hip Fracture Registry [32]. Our results are specifically applicable to patients with preserved cognitive function and partial functional independence at baseline, who have the potential to engage in mobility programs based on step count targets. Further research is needed to define appropriate mobility goals for more severely impaired populations.

Fourth, patient inclusion was lower than expected in the study period, mainly due to the high incidence of older institutionalized patients with significant functional and cognitive dependence, the exclusion of patients requiring four to six weeks of immobilization after surgery, and the impact of the COVID-19 pandemic.

Finally, the observational nature of the study precludes any causal inference regarding the relationship between step counts and functional recovery.

Further studies are necessary to validate the preliminary step count thresholds identified in this study across larger and more diverse cohorts and to refine activity goals tailored to different levels of baseline function in older adults after hip fracture.

## 6. Conclusions

In older adults discharged after hip fracture, our findings suggest that achieving more than 150 steps per day in the first month, 425 steps per day in the third month, and 2404 steps per day by the sixth month is associated with functional improvement of at least five points on the Barthel Index.

These preliminary step count targets may represent attainable and clinically meaningful goals that could support patient counseling and discharge strategies aimed at optimizing functional recovery. These thresholds, although exploratory, may also assist clinicians in monitoring and identifying patients at risk of poor functional recovery. Further research is needed to validate these thresholds in larger and more diverse populations, and prospective validation cohorts is needed to confirm these preliminary findings and to better define step count goals for functional recovery after hip fracture.

## Figures and Tables

**Table 1:** Baseline characteristics.

Characteristic	Included Patients (n = 94)		Withdrawn Patients (n = 50)		*p* Value
Gender					
Male	18	19.2%	18	36.0%	
Female	76	80.8%	32	64.0%	0.026
Age	85.04 ± 5.5		83.25 ± 6.0		0.084
Place of living					
Home	89	94.7%	48	96.0%	
Nursing home	5	5.3%	2	4.0%	0.726
Functional Barthel Index					
0–20	0	0%	0	0%	
21–60	0	0%	2	4%	
61–90	27	28.7%	14	28%	
91–95	10	10.6%	9	18%	
100	57	60.6%	25	50%	0.127
Pfeiffer Test					
No Cognitive Impairment	77	88.5%	40	90.9%	
Mild Cognitive Impairment	6	6.9%	4	9.1%	
Moderate Dementia	4	4.6%	0	0%	0.329
ASA Category					
II	38	40.9%	15	30.0%	
III	38	40.9%	23	46.0%	
IV	3	3.2%	4	8.0%	
Unknown	14	15.0%	8	16.0%	0.429
Type of fracture					
Intracapsular	40	42.6%	23	46.0%	
Intertrochanteric	46	48.9%	24	48.0%	
Subtrochanteric	6	6.4%	3	6.0%	
Basicervical	2	2.1%	0	0%	0.802
Side of fracture					
Left	56	59.6%	27	54.0%	
Right	38	40.4%	23	46.0%	0.519
Types of anesthesia					
General	8	8.5%	3	6.0%	
Neuraxial	84	89.4%	47	94.0%	
Others	2	2.1%	0	0%	0.703
FRAIL scale					
Frail	80	88.9%	41	93.2%	
Fit	10	11.1%	3	6.8%	0.431

n (%): Pearson’s chi-square test. Median (p25; p75): Mann–Whitney-U Test.

## Data Availability

The original contributions presented in this study are included in the article. Further inquiries can be directed to the corresponding author.
